# The Relationship between Change of Direction Tests in Elite Youth Soccer Players

**DOI:** 10.3390/sports7050111

**Published:** 2019-05-14

**Authors:** Björn Kadlubowski, Michael Keiner, Hagen Hartmann, Klaus Wirth, Ulrich Frick

**Affiliations:** 1 Department of Sport Science, Germany University of Health & Sport, 85737 Ismaning, Germany; michaelkeiner@gmx.de (M.K.); hagen-hartmann@online.de (H.H.); 2 DSC Arminia Bielefeld e. V, 33615 Bielefeld, Germany; 3 Sport and Exercise Sciences, University of Applied Sciences, 2700 Wiener Neustadt, Austria; k.wirth@fhwn.ac.at; 4 Institute of sport science, Johann Wolfgang Goethe-University, 60323 Frankfurt/Main, Germany; u.frick@sport.uni-frankfurt.de

**Keywords:** 505, COD performance, COD tests, Gewandtheitslauf, Illinois agility test, soccer players, square test, T test, triangle test

## Abstract

Change of direction (COD) is a performance-limiting factor in team sports. However, there are no exact definitions describing which physical abilities limit COD performance in soccer. Nevertheless, different COD tests are used or have been recommended as being equally effective in the professional practice of measuring COD performance. Therefore, the aim of this study was to evaluate the relationship between different COD tests, and to test the independence and generalizability of these COD tests in soccer. As such, 27 elite youth soccer players were randomly recruited and were tested in different COD tests (i.e., Illinois agility test (IAT), T agility test (TT), 505 agility test (505), Gewandtheitslauf (GewT), triangle test (Tri-t), and square test (SQT)). Bivariate Pearson correlation analysis was used to assess the relationships between the COD tests. The Benjamini–Hochberg method was used to control for the false discovery rate of the study at 0.05. This investigation calculated explained variances of 10% to 55% between performances in the different COD tests. This suggested that the tests covered different aspects or task-specific characteristics of the COD. Therefore, coaches and sport scientists should review and select different tests with a logical validity, based on the requirement profiles of the corresponding sport.

## 1. Introduction

Change of direction (COD) is a performance-limiting factor, especially in team sports, and it has been identified in several test batteries related to elite sports [1]. Reilly and Williams [2] proposed that the Illinois agility test (IAT) is the most appropriate test for measuring the COD performance in team sports. In contrast, Nimphius et al. [3] pointed out that the IAT is not suitable for testing directional speed. Meanwhile, other authors recommend the T agility test (TT) as the most suitable method for testing COD performance [4], whereas others [5,6,7] recommend the IAT, the TT, and the 505 agility test (505) for evaluating COD performance in team sport athletes. Furthermore, COD tests that are designed like the triangle test (Tri-t), or COD tests that are based on the Tri-t although designed like the square test (SQT), are commonly used in team sport assessments [8]. The German Soccer Association (DFB) recommends the Gewandtheitslauf (GewT) test to evaluate the COD performance of soccer players [9]. It is important to note that the Tri-t, SQT, and GewT have yet to be compared to other COD tests.

Different COD tests possess different durations, lengths, or degrees of COD [10] (Table 1). The different test designs (distance, number of CODs, etc.) indicate that the test performance is limited by different parameters. Some tests are limited by metabolic capacity, speed-endurance, and longer distances [11], while others are limited by the eccentric–concentric strength or power [7,12] or by the linear sprint performance [13].

In the literature, there are no exact definitions of which physical abilities limit performance during COD tests [8,17]. Nevertheless, different methods have been used or recommended as being equally effective in professional practice [5,18]. Research has shown an explained variance of 61% to 79% between the 505 and IAT tests (r = 0.78 to 0.89, *p* < 0.01) [18], while Raya et al. [19] showed an explained variance of 56% between the TT and the IAT (r = 0.75, *p* < 0.001). Furthermore, a study by Cinarli et al. [20] on the TT showed an explained variance of 0% to 13% between the IAT, 505 test, and GewT test (−0.053–0.365; *p* = 0.164–0.846), and an explained variance of 26% between the 505 and IAT tests (r = 0.52; *p* = 0.041). These results are supported by Hachana et al. [21], who also found an explained variance of 9% between the IAT and the 505 tests (r = 0.31; *p* = 0.002), and Draper and Lancaster [13], who show an explained variance of 6% between the IAT and the 505 tests (r = 0.25; *p* < 0.05). These studies were not performed using elite soccer players, but rather with moderately trained athletes. In general, the data stemming from studies using moderately trained subjects do not reflect the data of athletes that have been training for several years (i.e., high-performance athletes). The potential heterogeneous performance between the moderately trained participants suggests that statistically higher correlations may potentially overestimate the true correlation. The data from these investigations should therefore be handled with caution when drawing conclusions relating to elite athletes.

Consequently, the aim of this study was to evaluate the relationship between different COD tests, as well as to test the independence and generalizability of the COD tests, in elite soccer players. Moreover, we propose that moderate to strong correlations would only be revealed between tests that share a similar requirement profile (e.g., similar total test distance). On the other hand, tests with a heterogeneous requirement profile (e.g., heterogeneity in total test distance) should produce weak correlation coefficients. If the results indicate a generalizability of the COD tests, and deny a task specificity, COD tests could be used in soccer without knowledge of the specific requirements of the soccer game for direction changes and vice versa. This information may benefit coaches and sports scientists in the design of COD tests during their professional practice.

## 2. Materials and Methods

To evaluate this research question, 27 elite youth soccer players from the training center of a professional soccer club in the 2. Bundesliga (Germany) were recruited. The soccer players were tested using the TT, IAT, 505, GewT, Tri-t, and SQT tests.

### 2.1. Subjects

We randomly recruited 27 soccer players (male; age: 18.5 ± 4.5 years.; height: 1.81 ± 0.17 m; weight: 76.2 ± 17.7 kg) from the under 17 years old (U-17), under 19 years old (U-19), and under 23 years old (U-23) teams of the participating training center. These youth soccer teams played in the highest German junior divisions (i.e., the U-17 Bundesliga and the U-19 Bundesliga). The soccer players were considered highly trained, with four soccer training sessions per week, and they competed on the weekend. All participants had played soccer since early childhood and, therefore, were highly trained relative to their age. The participants had not engaged in fatiguing training sessions for a minimum of 3 days before going through the testing. None of the participants reported any injuries at the time of testing.

Each participant and his parents (if the participant was younger than 18 years old) were informed of the experimental risks involved with the research. All participants and their parents (if the participant was not 18 years old) provided written informed consent to participate in the present study. Approval for this study was obtained from the institutional review board at the German University of Health and Sport (No. 01/2019.92002800). The study was performed with the use of human participants in accordance with the Helsinki declaration.

### 2.2. Procedures

**Figure 1:**
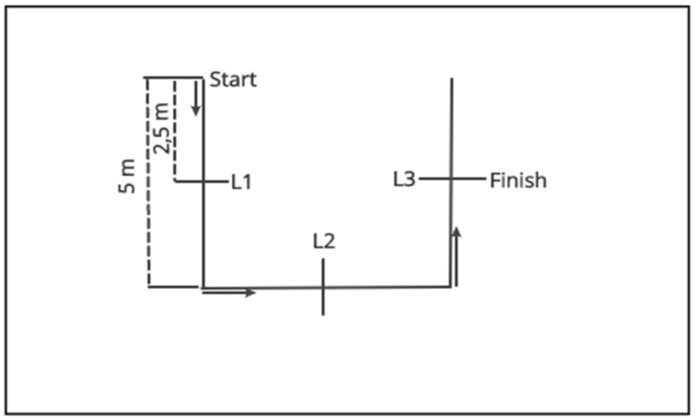
Structure and dimensions of the newly developed change of direction (COD) test. L1–3, timing gates 1–3.

The soccer players were tested using the TT, IAT, 505, GewT, Tri-t, and SQT tests. The descriptions of the test setups can be found in the literature as follows: 505 [10], TT [22], IAT [23], GewT [9], and Tri-t [8]. The SQT consisted of two vertical sections and one horizontal section, each with a length of 5 meters and each with timing gates set at 2.5 meters. Timing commenced at the first start of the barrier, followed by measurements at intervals of 2.5 meters, 7.5 meters, and 12.5 meters. The test course was square, and it included two 90 degree turns (Figure 1).

The six tests were between 10 and 36.6 meters in length (Table 1). The data were assessed during the soccer season, and three days after a championship match. The data were measured on two different testing days, that were separated by one week to avoid distortions from fatiguing effects. Three test procedures were run on any one testing day.

The performance of the test procedures was divided into two days, separated by one week between them. The order of the test procedures on testing days 1 or 2 was randomly determined at the beginning of the study. The tests were performed on the testing day based on the total distance in ascending order, in order to avoid fatiguing effects. On testing day 1, the TT, 505, and IAT were performed, and on testing day 2, the Tri-t, SQT, and GewT were performed, in the described order. The subjects were allowed three attempts per test, which were separated by a 3 min break. The best attempt was used for the statistical analysis. If the pylons or hurdle bars were knocked down or touched during testing, a follow-up run was then completed. The tests were separated by a break of 20 min. The timing was measured within all the different tests using a double-timing gate system (Browser TC Timing System, Biederitz, Germany). The starting point was marked with a small cap 0.75 meters away from the starting gate to avoid early triggering, e.g., by a hand movement or a bent body position.

The warm-up was standardized for each testing day. The warm-up consisted of nonspecific running at low-to-medium intensity for approximately 5 min. Then, coordination exercises, such as running with the knees lifted, heeling, and side steps, were performed for approximately 5 min. Next, the athletes completed a 5-min dynamic stretching program (standing scales, hand walks, lunge steps with twisting, and lateral lunges with rotation). Subsequently, three acceleration runs over approximately 30 m (meters) were performed with short intervening walking breaks. The total warm-up time on each test day was 20 min.

### 2.3. Statistical Analyses

The SPSS 25.1 program (IBM, Ehningen, DE, Germany) was used for the statistical data analysis. The significance level for all the statistical tests was set a priori at *p* < 0.05. The normality of the data distribution was tested using the Kolmogorov–Smirnov test. Data were expressed as the mean ± SD. Reliability analyses were performed using the intraclass correlation coefficient (ICC) and a 95% confidence limit as per Shrout and Fleiss [24]. For the reliability analyses, all three measured trials were analyzed. Furthermore, a bivariate Pearson correlation analysis was used to assess the relationship between the different COD tests. The best time of each test was used for the statistical analysis. If the data were not normally distributed, the relationships between the test variables were then calculated using Spearman’s correlation coefficient. The relationships were classified as follows: 0 = no correlation, 0 < |r| < 0.2 = very weak correlation, 0.2 ≤ |r| < 0.4 = weak correlation, 0.4 ≤ |r| < 0.6 = moderate correlation, 0.6 ≤ |r| < 0.8 = strong correlation, 0.8 ≤ |r| < 1.0 = very strong correlation, and 1 = perfect correlation, as described by Bortz and Doring [25]. The determination coefficient (r^2^) was calculated by squaring the correlation coefficient (r). The Benjamini–Hochberg method was used to control the false discovery rate of the study at 0.05 as in Reference [26].

## 3. Results

The COD performances of all the different tests are displayed in Table 2. All the parameters were normally distributed. 

The intraclass correlation coefficients (ICC) and the 95 percent confidence intervals (95% CIs) of the performance tests are displayed in Table 3. The ICCs were above a coefficient of r = 0.9. This level is classified as excellent, as described in reference [27]. This excluded the SQT-L, which had an ICC of r = 0.88, and the test with the shortest average time (the 505), which had an ICC of r = 0.72. The test with the longest average mean time (the IAT) had an ICC of r = 0.94.

The Pearson product–moment correlation coefficients between the different COD tests are shown in Table 4. After corrections using the false discovery rate (FDR), the highest *p*-value for a significant correlation was r = 0.027. The *p*-values corrected using an FDR of 0.05 are shown in Table 4. The highest significant correlation was calculated between the SQT-L and the Tri-t-R (r = 0.74), as well as between the Tri-t-L and the Trit-R (r = 0.63). Meanwhile, the least significant coefficient was between the Tri-t-L and the IAT (r = 0.42).

## 4. Discussion

The aim of this study was to evaluate the relationship between different COD tests, as well as to test the independence and generalizability of the COD tests, in elite soccer players. This investigation calculated explained variances of 8% to 55% (a range of weak to strong correlation coefficients) between the performances achieved in different COD tests. Strong significant correlations were calculated from similar test designs, such as the SQT-L and the Tri-t-R (r = 0.74; r^2^ = 0.55), as well as between the Tri-t-L and Trit-R (r = 0.63; r^2^ = 0.40). However, this was not a stringent observation, because the SQT-R and the Tri-t-R were only correlated in a weak manner (r = 0.33; r^2^ = 0.11). This suggested that even similar test designs covered different aspects or task-specific characteristics of the COD [28]. The generalizability of COD tests must therefore be denied.

The results of this investigation were in line with the results in the current literature that also showed heterogeneity in the explained variances (0% to 79%) between different COD tests. In general, this range is explained by different study designs, using subjects from different sports, and training levels. Hachana and Chaabène [21] examined 105 male sports students who played sports, such as rugby, handball, or soccer. They presented a weak correlation (r = 0.32; *p* = 0.02) and an explained variance of 9%. In contrast, Raya et al. [19] showed a strong correlation between the TT and the IAT (r = 0.75; *p* < 0.01), and an explained variance of 56%. The study analyzed 97 soldiers and recorded the test results within a period of 48 hours. The difference in the calculated correlation coefficients may have been due to the more homogeneous performance level of the participants in this study, which leveled out the potential overestimation of the real correlation coefficients (r = 0.29; r^2^ = 0.08). This fact might also explain the different correlation coefficients in the TT and 505 results from this study and from the research of Stewart and Turner [18] (r = 0.38; r^2^ = 0.14; *p* > 0.05 versus r = 0.81; r^2^ = 0.66; *p* < 0.05).

Nevertheless, these results conformed to the heterogeneity of the explained variances between the different COD tests, although they assumed that there was a task-specific characteristic in the COD tests. This task-specific characteristic can, for example, be assumed from the explained variance between the IAT and Trit-L/Trit-R tests (5% and 18%, respectively) in this study, as explained by the different structures of the tests (Table 1). Owing to the different test structures, the tested parameters might vary (e.g., speed or speed towards the COD). Therefore, the performances under different tests might be limited by the different parameters, such as metabolic capacity [11], while other tests might be limited by the eccentric–concentric strength or power [7,12], or by the linear sprint performances [13]. Consequently, the highest explained variance might be assumed to be associated with the most similar test structures, as was the case in the Tri-t-R and Tri-t-L or for the SQT-L and SQT-R. Both tests only varied in the direction of rotation, where the explained variances were calculated as 26% and 27%, respectively. This might be explained by the lateral differences in leg strength, because research has demonstrated the influence of the dominant versus non-dominant leg on performance in different directions [29,30]. This lateral difference might be strengthened by constantly repeating the use of the dominant turning side during the soccer match and training [31,32], and it might have resulted in different performances in the Trit-L and Trit-R tests (3.10 ± 0.12 s versus 3.14 ± 0.13 s, respectively). However, this effect was limited in the SQT-L and SQT-R tests (3.19 ± 0.15 s versus 3.21 ± 0.13 s, respectively). Nevertheless, the explained variance of this study may also derive task-specific characteristics, even with small differences in the test structure (i.e., degree/direction of rotation). This rationale seemed to apply not only to the shortest tests, but also to the longest tests (IAT and TT; variance explanation: 8%).

However, the highest explained variance in this study was found between the SQT-L and Tri-t-L tests (i.e., a 55% explained variance between the two tests). Although both tests had a minimal difference in the distance of 2.5 m and in the degree of COD, they were similar in the number of direction changes and the direction of rotation. This explained variance (the highest measured in this study) could indicate that the direction of rotation or the number of CODs had a greater influence on the explanatory rate than on the degree of the change of direction or exact length of the test. We also have to consider whether the majority (confirmed) of the players are right-footed and have a better left rotation as a result. Nevertheless, even the highest explained variance (r^2^ = 0.55, *p* < 0.05, for the FDR of this study) was far from the level required to assume generalizability of the COD tests in team sports [33].

However, by adjusting the *p*-values that were originally significant in this study for the FDR, some values missed the significance level. It should be noted that a hypothetical probabilistic problem may be corrected due to this correction. Therefore, based on the original significant *p*-values, also citing caution, the influence of these variables cannot be completely denied. It is also important to point out that the ICC of 505 was lower than that of the other tests, and, therefore, the calculations from 505 should be considered with caution. Nevertheless, this investigation calculated explained variances of 10% to 55% between the performances of the different COD tests. This suggested that the tests covered different aspects or task-specific characteristics of the specific CODs [26].

## 5. Conclusions

Data from this investigation assumed that the COD tests measured task-specific performances. Therefore, coaches and sport scientists must review and select different tests with logical validity, based on the requirement profiles of the corresponding sport. During a soccer match, sprints with a duration of 2 to 4 seconds occur almost every 90 seconds [34]. Moreover, 83.9% of changes of direction in soccer games are between 0 and 90° [35]. Given the requirements of professional soccer games, the square and triangle tests could be used to test the COD performance, although further investigations would be necessary in the future to identify the appropriate COD test in terms of the maximum representation of the CODs linked to a specific sport.

## Figures and Tables

**Table 1:** Requirements of the different change of direction tests.

Test	Total Test Distance (m)	Number of CODs	Degree of COD	Approximate Total Time of the COD Test (s)	References
Triangle test	10	2	60°	2.9–3.5	[8]
Square test	12.5	2	90°	2.9–3.5	Figure 1
505 agility test	20	1	180°	2.0–3.0	[14]
Gewandtheitslauf	20	6	90°	7–8	[11]
T agility test	36.6	4	90°	10–12	[7,15]
Illinois agility test	36.6	9	90°, 180°	14–18	[12,15,16]

COD, change of direction; m, meter; s, seconds.

**Table 2:** The change of direction performance results.

Test	Mean ± SD (in s)
505	2.17 ± 0.06
IAT	13.76 ± 0.31
TT	9.21 ± 0.30
GewT	7.50 ± 0.36
Tri-t-L	3.10 ± 0.12
Tri-t-R	3.14 ± 0.13
SQT-L	3.19 ± 0.15
SQT-R	3.21 ± 0.13

TT, T agility test; 505, 505 agility test; IAT, Illinois agility test; GewT, Gewandtheitslauf test; Tri-t-R, triangle test right around; Tri-t-L, triangle test left around; SQT-L, square test left around; and SQT-R, square test right around.

**Table 3:** The reliability analyses results.

Reliability Analyses	TT	505	IAT	GewT	Tri-t-L	Tri-t-R	SQT-L	SQT-R
**ICC**	0.90	0.72	0.94	0.93	0.92	0.93	0.88	0.93
**95% Cl**	0.81–0.95	0.48–0.87	0.88–0.97	0.88–0.96	0.88–0.95	0.9–0.96	0.82–0.93	0.89–0.95

ICC, intraclass correlation; 95% Cl, ±95% confidence interval; TT, T agility test; 505, 505 agility test; IAT, Illinois agility test; GewT, Gewandtheitslauf test; Tri-t-R, triangle test right around; Tri-t-L, triangle test left around; SQT-L, square test left around; and SQT-R, square test right around.

**Table 4:** Pearson product–moment correlation coefficients for the change of direction (COD) tests.

Tests		TT	505	IAT	GewT	Tri-t-L	Tri-t-R	SQT-L	SQT-R
**TT**		/							
**505**	r	0.38	/						
	r^2^	0.14							
**IAT**	r	0.29	0.51 *	/					
	r^2^	0.08	0.26 *						
**GewT**	r	0.31	0.36 #	0.61 *#	/				
	r^2^	0.10	0.13 #	0.37 *#					
**Tri-t-L**	r	0.63 *#	0.45 *#	0.42 *	0.51 *#	/			
	r^2^	0.40 *#	0.20 *#	0.18 *	0.26 *#				
**Tri-t-R**	r	0.63 *#	0.43	0.23	0.37	0.51 *#	/		
	r^2^	0.40 *#	0.18	0.05	0.14	0.26 *#			
**SQT-L**	r	0.60 *#	0.44 *	0.32	0.53 *	0.74 *#	0.51 *	/	
	r^2^	0.36 *#	0.19 *	0.10	0.28 *	0.55 *#	0.26 *		
**SQT-R**	r	0.61 *#	0.45 *	0.47 *#	0.56 *#	0.46 *#	0.33 #	0.52 *#	/
	r^2^	0.37 *#	0.20 *	0.22 *#	0.31 *#	0.21 *#	0.11 #	0.27 *#	

TT, T agility test; 505, 505 agility test; IAT, Illinois agility test; GewT, Gewandtheitslauf test; Tri-t-L, triangle test left around (measurement after 10 meters); Tri-t-R, triangle test right around (measurement after 10 meters); SQT-L, square test left around (measurement after 12.5 meters); SQT-R, square test right around (measurement after 12.5 meters); * level of significance (*p* < 0.05) and # *p* < false discovery rate of 0.05 for this study.
